# First person – Wei-Lun Hsu

**DOI:** 10.1242/dmm.050552

**Published:** 2023-11-09

## Abstract

First Person is a series of interviews with the first authors of a selection of papers published in Disease Models & Mechanisms, helping researchers promote themselves alongside their papers. Wei-Lun Hsu is first author on ‘ High-fat diet induces C-reactive protein secretion, promoting lung adenocarcinoma via immune microenvironment modulation’, published in DMM. Wei-Lun is a PhD student in the lab of Kang-Yi Su at National Taiwan University, Taipei, Taiwan, investigating possible treatments for obesity-related diseases and obesity.


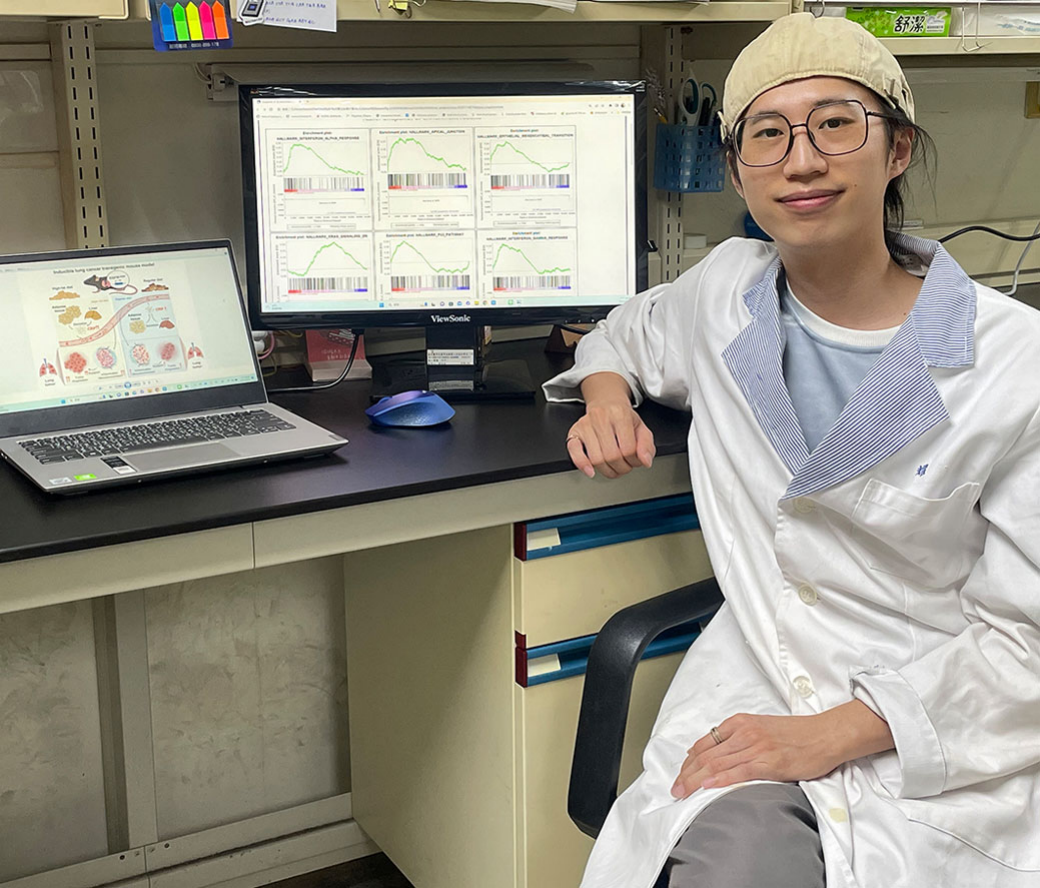


**Wei-Lun Hsu**

**How would you explain the main findings of your paper to non-scientific family and friends?**

Lung cancer is among the most prevalent cancer types worldwide. In this study, we used a mouse model to induce lung cancer specifically and fed these mice different diets to see if the severity of lung cancer differed. We found that the high-fat diet (HFD) created a different immune environment in the lungs and increased lung tumor cell proliferation compared to the regular diet (RD). We discovered that an immune factor called C-reactive protein (CRP) was increased due to elevated production by the liver and adipose tissue under HFD stimulation. CRP circulated to the lungs in the blood and could directly enhance the development of lung tumors. So, according to our study, consuming too much high-fat food can contribute to lung cancer development. Moreover, if we find a way to decrease CRP levels when treating lung cancer, it may potentially slow down the progression of lung cancer.

[…] according to our study, consuming too much high-fat food can contribute to lung cancer development.

**What are the potential implications of these results for your field of research?**

In our research model, we explored the impact of the HFD on lung cancer. By integrating histological analysis with high-throughput data, we found that mutant EGFR-driven transgenic mice fed an HFD exhibited more robust immune responses in their lungs because of significant NF-κB upregulation. This suggests that the HFD influences the immune microenvironment in lung cancer and promotes tumor growth. We uploaded available lung tissue microarray data under two dietary conditions in the NCBI Gene Expression Omnibus database. These data can further promote a deeper understanding of the relationship between the cancer immune microenvironment and tumor growth. Also, we performed immune-related cytokine arrays and obesity-related adipokine arrays. We believe that these resources will facilitate and help various laboratories explore the effects of different diets on lung cancer development. Researchers can attempt to discover potential targets, such as CRP, or develop some combination therapies to suppress lung cancer after an advanced conjoint analysis with our data in the future.

**What are the main advantages and drawbacks of the experimental system you have used as it relates to the disease you are investigating?**

We utilized the ‘Tet-on’ system in mutant EGFR-driven mice models to research the impact of the HFD on lung cancer. It is easy for researchers to manipulate the timing of inducing lung cancer by feeding mice different diets containing doxycycline. Moreover, this model can mitigate many confounding factors, such as the possible synergistic effects of injecting lung cancer-inducing drugs or human errors in handling. It provides a more convincing basis for discussing the varied cancer developments resulting from different diets. However, there still exist drawbacks to our model. Our model is restricted to assessing whether the HFD causes similar results in other major factors leading to lung cancer, such as K-RAS mutation. Our model's limited representativeness reflects the need for further discussion and research in the future.

**Figure:**
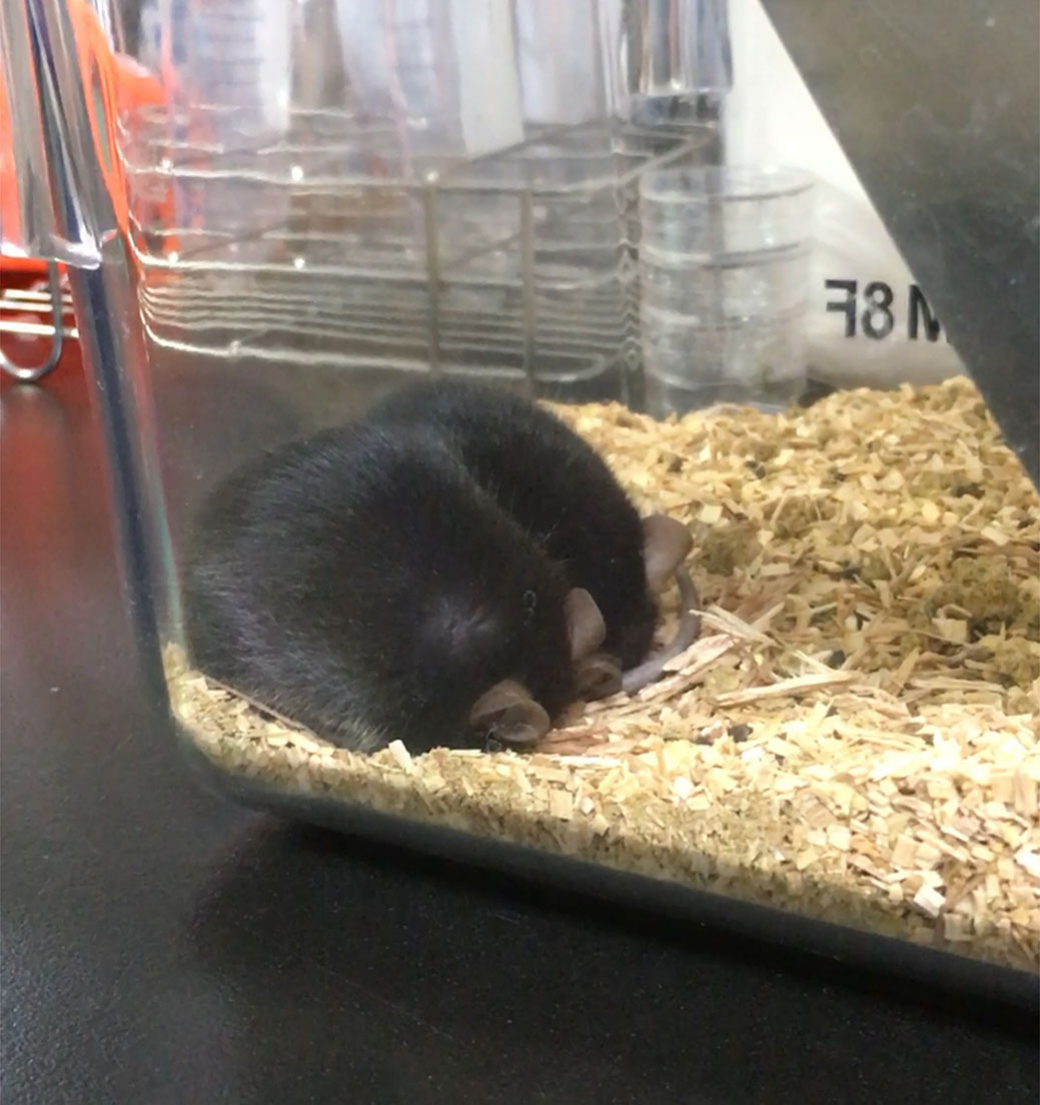
Mutant EGFR-driven transgenic mice administered different diets and used to understand the effects of obesity on lung cancer development.

**What has surprised you the most while conducting your research?**

In the experimental mice fed the different diets containing doxycycline, the high-fat group initially showed an upward trend in body weight. However, surprisingly, their weight decreased instead of continuing to rise in the later stages of the experiment. While we did not delve into the underlying reasons and attributed it to tumorigenesis, this phenomenon could be a potential avenue for future research. Exploring this phenomenon might enhance our understanding of the impact of different diets on lung cancer and offer insights into how metabolic changes occur when diet-induced lung cancer develops.

**What do think is the most significant challenge impacting your research at this time and how will this be addressed over the next 10 years?**

We still have a limited understanding of the connections between cancer development and various metabolic processes within the body. Curing cancer is a challenge, so finding a balance that allows us to co-exist with cancer, without succumbing to it, could be required. This approach could prevent cancer from proliferating while sustaining our survival and life quality. Achieving this delicate balance will require continuous research and exploration. Investigating the relationship between body metabolism and cancer growth might be a way to help us co-exist with cancer until a perfect treatment is discovered in the future.

**What changes do you think could improve the professional lives of scientists?**

In my opinion, different countries, research institutions, schools and units focusing on life sciences can often host numerous conferences, enabling scientists from various fields worldwide to share and absorb the latest academic knowledge. Furthermore, if society can support and create a friendly research environment for scientists, providing them with achievable goals, it will undoubtedly help scholars to devote themselves to their research pursuits.

[…] if society can support and create a friendly research environment for scientists, providing them with achievable goals, it will undoubtedly help scholars to devote themselves to their research pursuits.

**What's next for you?**

My ongoing goal is to continue to understand and research diseases related to obesity, enhancing my knowledge in this field. I hope to contribute to the academic understanding of obesity-related issues in the future. But, of course, for my immediate goal, I'll focus on graduating from my PhD program.
